# A dynamic subset of network interactions underlies tuning to natural movements in marmoset sensorimotor cortex

**DOI:** 10.21203/rs.3.rs-3750312/v1

**Published:** 2023-12-27

**Authors:** Nicholas Hatsopoulos, Dalton Moore, Jason MacLean, Jeffrey Walker

**Affiliations:** University of Chicago; University of Chicago; University of Chicago; University of Chicago

## Abstract

Mechanisms of computation in sensorimotor cortex must be flexible and robust to support skilled motor behavior. Patterns of neuronal coactivity emerge as a result of computational processes. Pairwise spike-time statistical relationships, across the population, can be summarized as a functional network (FN) which retains single-unit properties. We record populations of single-unit neural activity in forelimb sensorimotor cortex during prey-capture and spontaneous behavior and use an encoding model incorporating kinematic trajectories and network features to predict single-unit activity during forelimb movements. The contribution of network features depends on structured connectivity within strongly connected functional groups. We identify a context-specific functional group that is highly tuned to kinematics and reorganizes its connectivity between spontaneous and prey-capture movements. In the remaining context-invariant group, interactions are comparatively stable across behaviors and units are less tuned to kinematics. This suggests different roles in producing natural forelimb movements and contextualizes single-unit tuning properties within population dynamics.

## Introduction

The study of the relationship between population activity in primary motor cortex (M1) and upper limb motor behavior has taken one of two diverging approaches. On the one hand, single neuron tuning properties have been characterized and then combined across the population without considering the interactions between neurons^1,2^. On the other hand, low-dimensional structure in population dynamics has been linked to movement but omits details of single neuron tuning properties^3,4^. Although these divergent methodologies have each proven useful, comparatively few studies have attempted to place single neuron tuning properties in the context of broader M1 population dynamics. Pairwise spike count correlations provide information about motor behavior beyond what is provided by firing rates alone^5^ and improve encoding models that predict single-unit activity^6^. Recent work has also demonstrated that pairwise spike time correlations carry behaviorally relevant information in M1^7^. A large body of work in mostly primary visual cortex has shown that recurrent interactions within local functional groups can explain single-trial neural activity^8^, that both tuned and untuned units are essential components of the functional network (FN)^9^, and suggested that reliable pairwise correlations, rather than first-order statistical features of spike trains, are the building blocks of coding in visual cortex^10^.

Our understanding of single-unit activity in the context of neuronal interactions has been limited in many cases by the necessity for trial averaging, by restrained and constrained movements due to wired neural recording systems, by the difficulty in quantifying complex kinematics, or by the constraints of analysis methods. Constrained and over-trained behavioral tasks such as center-out reaching or pedaling a wheel^11,12^ limit the variability in all movement parameters aside from those under investigation and may impose an artificial ceiling on the dimensionality of neural population dynamics^13^. Even in the case of less stereotyped behaviors like random target pursuit^14^, reaching around obstacles^15^, and reach to grasp tasks^16^, primates are restrained in a chair with head-fixation and require weeks of daily training to reliably execute movements. There is evidence to suggest that a significant portion of M1 neurons encode a constrained, well-trained task differently than free behavior^17^ and that over-training increases synchrony between M1 neurons^18^. The extent to which findings discovered in the constrained, trained setting will generalize to natural movements remains unclear.

Here we use a wireless neural recording system and computer-vision pose estimation tools to study unrestrained and untrained arm movements executed by the common marmoset (*Callithrix jacchus*) during prey capture of moths^19–22^. Compared to classical task paradigms, this behavior produces a richer set of movements with variable directions, speeds, and amplitudes and obviates concerns related to overtraining. Instead of assuming a fixed time lag between neural activity and kinematic features^11,23–29^, we quantify single unit tuning properties using a temporally extended trajectory tuning model^14^. At the same time, we incorporate neuronal interactions using a functional network (FN) that describes pairwise spike time statistics in the form of a weighted, directed graph. We show that the trajectory tuning model extends to more naturalistic movements and explains neural activity better than a simpler model. We demonstrate that tuning to kinematics depends on the functional interactions between units – particularly on structured strong connections. Finally, we identify a context-specific functional group that reorganizes during natural forelimb movements necessary for prey capture. This context-specific functional group is strongly interconnected and comprises units tightly linked to kinematics with strong, positively correlated preferred trajectories.

## Results

**Figure 1.**
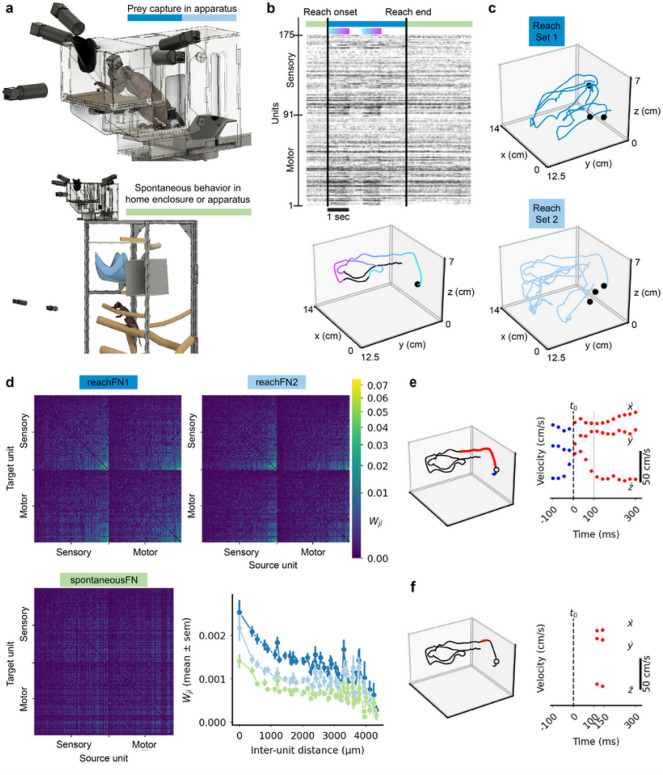
Behavior, data collection and encoding model construction. (A) *Top:* subject capturing prey in the apparatus Behavioral video data was collected by five (MG) or two (TY) cameras *Bottom:* subject behaving freely and spontaneously in the home enclosure. (B) *Top.* A raster plot shows the activity of 175 units recorded from TY. from one second before to 3 seconds after the duration of reach 3. Units are ordered as in C. The green and blue bars above the raster indicate times corresponding to spontaneous behavior (green) or prey capture (blue). The gradient bars highlight penods of increased firing rates across the population. *Bottom:* hand position for reach 3. with reach onset indicated by the black dot and the same gradient indicating neural modulation imposed on the kinematics. (C) A sample of hand trajectories for six of 101 reaches, separated into reachSetl (dark *blue)* and reachSet2 *(light blue).* (D) FNs were computed from activity during either reaches in the corresponding reachSet or during spontaneous behavior Within motor or sensory areas, units are ordered by the average in-weight to the target unit for reachFN1/2. The color scale corresponds to $w_{ji}\;=\;conMI$ *Lower Right:* Functional weights versus inter-electrode distance. (E) A representative sample for the trajectory and full kinematics models with $\tau_{\text{lead}}=100\;ms$ and $\tau_{lag}=300\;ms$*. Lett,* wrist position for a single reach *(black)* overlaid with the spike sample time *(white circle)* and corresponding trajectory sample including lead *(blue)* and lag *(red)* movements subsampled at 40 Hz. *Right,* velocity samples with the spike sample window and trajectory center shown with a vertical black dashed or dotted line, respectively. (F) The corresponding sample for the velocity and short kinematics models with $\tau_{lag1}=100\;ms$ and $\tau_{lag2}=150\;ms$..

Each marmoset was allowed to voluntarily enter and exit the prey-capture apparatus attached to the top of the home enclosure (Fig. 1a). Movement was recorded by high-speed cameras that were automatically triggered using an infrared beam beam-breaker switch (see Methods). Spontaneous behavior in the home enclosure was recorded continuously by a separate camera system, although we do not quantify kinematics or report classes of spontaneous behavior in this work. Spiking activity was recorded throughout the recording session (TY: 175 units, Fig. 1b; MG: 73 units, Supplementary Fig. 1a), during which the monkey was actively reaching for prey (TY: 101 reaches, MG: 56 reaches) or engaged in undirected, spontaneous behavior. We observed clear modulation of M1 and somatosensory cortical (S1) neurons (see Methods for estimation of the boundary between areas); spike rates increased across much of the population during extension of the hand and decreased during movements back toward the partition (Fig. 1b, Supplementary Fig. 1a). Reaches were randomly assigned to *reachSet1* or *reachSet2* and the corresponding spiking activity during reaches and during spontaneous behavior was used to compute functional networks denoted *reachFN1*, *reachFN2*, and *spontaneousFN* (Fig. 1c–d; Supplementary Fig. 1b-c). Functional networks were computed using the confluent mutual information (conMI) between the binned spike trains of each neuron pair^30^ (see Methods). The structures of reachFN1 and reachFN2 were qualitatively indistinguishable in the connection matrices; both exhibited strong same-electrode connectivity and gradually decreasing weights with increasing inter-electrode distance. The structure of connectivity was different in the spontaneousFN for both monkeys with depressed weights at shorter inter-electrode distances – especially for same-electrode functional connections (Fig. 1d, Supplementary Fig. 1c). We built generalized linear models (GLMs) to predict single-sample spiking activity of individual units given a temporally-extended hand velocity trajectory (i.e., velocities that either led or lagged the spike sample) and average position of the hand throughout the trajectory, similar to previous work in macaques^14,16,31,32^. We refer to these sets of models with and without position terms as the *full kinematics* and *trajectory* model, respectively (Fig. 1e). We also built models that approached a time-independent preferred direction representation by sampling brief velocity trajectories and average position between + 100ms and + 150ms with respect to the spike sampling time, which is often treated as the optimal lag between a motor cortical neuron’s firing and velocity^26,33^. We call these the *short kinematics* (brief trajectory and average position) and *velocity* (brief trajectory only) models (Fig. 1f).

### Encoding models incorporating temporally-extended velocity trajectories and average position predict single-unit spiking activity

**Figure 2:**
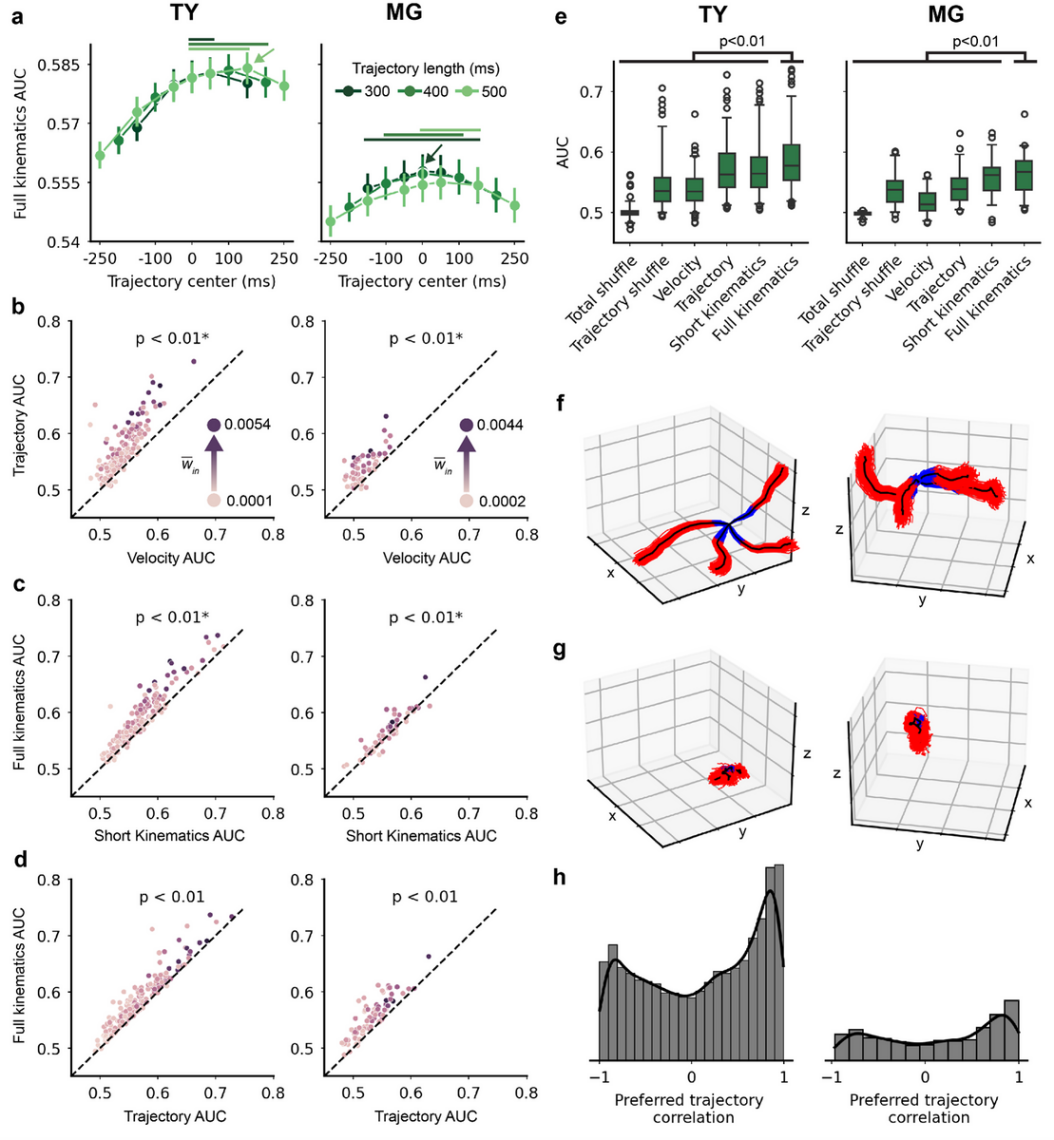
Full kinematics encoding model predicts single-unit activity. (A-H) *Left*: monkey TY. *Right*: monkey MG. (A) Mean ± sem AUC for the full kinematics model, all lead-lag sets. The trajectory models range from 300ms to 500ms in duration, and from lead-heavy samples on the left to lag-heavy samples on the right. The arrow indicates the best-performing lead-lag set. Solid lines above the data denote lead-lag sets for which AUC distributions were not significantly different than the best model (p>0.05, one-sided sign test with Bonferroni correction). (B) Scatterplot of the trajectory and velocity model AUC values for each unit, averaged over 500 train/test splits. Each unit’s hue corresponds to average in-weight in the FN, which will be explained in further detail in Fig. 3. Units above the unity line were predicted better by the trajectory model. P-values are the result of a one-sided sign test. In B and C, the asterisk indicates that we filtered out units that were not significantly tuned to the trajectory, leaving 163/175 units for TY and 59/73 units for MG. The trajectory model predicted activity better than the velocity model (TY: p<0.01 by one-sided sign test; MG: p<0.01). (C) The full kinematics model predicted activity better than the short kinematics model (TY: p<0.01; MG: p<0.01). (D) The full kinematics model outperformed the trajectory model due to the inclusion of average position terms (TY: p<0.01; MG: p<0.01). (E) Summary of model performance. Whiskers incorporate the middle 95% of data, with circles for the 2.5% at each end of the distribution. The full kinematics model produced AUC values significantly higher than all other models (p<0.01). (F) The preferred trajectory pathlets for four units with high AUCs. Each of 500 train/test splits is shown in blue and red corresponding to lead and lag movements, and the average pathlet is shown in black. (G) The pathlets for four units with the lowest AUCs. (H) Histograms of the Pearson correlation between pathlets for all pairs of units.

For each unit in the population, we tested 17 sets of each encoding model with varying lead and lag times in the trajectory samples – the models were 300–500ms in duration and ranged from entirely lead to entirely lag kinematics. We evaluated all models using the area under the receiver operating characteristic curve (AUC) computed on held-out test data, a metric which ranges from ~ 0.5 (no predictive power) to 1.0 (perfect prediction). For each model and unit, we fit and tested 500 independent GLMs using a different train/test split. We found that the full kinematics model performed best when it incorporated both lead and lag kinematics, particularly for models containing majority lag kinematics (Fig. 2a). We report subsequent results for the [−100, + 300]ms model due to its high performance in both monkeys; we confirmed that results match qualitatively across multiple lead-lag sets with similar performance.

We created two shuffled models for comparison with real data - *total shuffle* and *trajectory shuffle*. For the total shuffle model we permuted the kinematic samples to break the relationship between kinematics and spikes. For the trajectory shuffle model we permuted just the trajectory samples, leaving the relationship between average position and spikes unperturbed. We assessed tuning to kinematics and to the trajectory specifically for individual units by comparing all train/test splits of the full kinematics model to paired total shuffle and trajectory shuffle samples, respectively. For monkey TY, 172 of 175 units were significantly tuned to full kinematics (p < 0.01, one-sided sign test with Bonferroni correction) and 163 units were tuned to the trajectory specifically (p < 0.01). For monkey MG, 71 of 73 units were tuned to full kinematics (p < 0.01) and 59 units were tuned to the trajectory (p < 0.01). For comparisons between models using the brief and full trajectories shown in Fig. 2b–c, we included only the units that were tuned to trajectory details.

Next we compared model performance at the population level. The trajectory encoding model predicted spikes more accurately than the velocity model (TY: p < 0.01, one-sided sign test; MG: p < 0.01; Fig. 2b) and the full kinematics model was more accurate than short kinematics (TY: p < 0.01; MG: p < 0.01; Fig. 2c), confirming previous studies and showing that activity of individual neurons is more closely related to temporally-extended velocity trajectories than to ballistic velocity at a fixed time. The full kinematics model was significantly more predictive than the trajectory model (TY: p < 0.01; MG: p < 0.01; Fig. 2d), as well as all the other kinematics models we tested (Fig. 2e).

We treated the coefficients of the velocity trajectory terms in the full kinematics model as instantaneous velocities and integrated to obtain the preferred position trajectory, or *pathlet*^14^. Units with high AUC values were tuned to high amplitude preferred pathlets that were consistent across train/test splits, while units with low AUC values had no discernible preferred pathlet (Fig. 2f–g). We computed the Pearson correlation between pathlets for all pairs of units and found that preferred pathlets tended to be strongly and positively correlated, with a smaller peak of strong negative correlations (Fig. 2h). The population was dominated by tuning to extension of the hand or, to a lesser extent, hand retraction (Supplementary Fig. 2a); this agrees with our observation of modulation related to extension on individual reaches and explains the distribution of correlations shown here. The full kinematics model incorporating a temporally-extended hand velocity trajectory and average position over the movement sample was the best predictor of single-unit spiking activity, and individual units exhibited distinct preferred trajectories that tended to be strongly correlated with a preference for positive correlations.

### Network features improve the encoding model and strong functional inputs correlate with kinematic tuning

We incorporated the FN in each target unit’s encoding model by taking the dot product of all source unit spikes with the edge weights from the source units to the target unit (Eq. 6) for the leading bin and the coincident bin - this resulted in two *network features* for each target unit model. Trajectory samples belonging to reachSet1 were paired with network features computed using edge weights from reachFN2, and vice versa. We did this to ensure that trajectory and network features were independent (did not co-vary) within a single sample. We created network features using the computed FN rather than the alternative - fitting all pairwise coupling coefficients in the GLM – for three reasons. First, using conMI values as the coupling coefficients to produce network features constrained the encoding models to strict dependence on finely timed spike-train statistics measured directly from the neural data rather than on maximum likelihood computations. Prior work has shown that couplings taken from an FN approached optimality for most units in a similar model^34^. Second, our approach allowed us to manipulate the FN directly and observe the effect on model performance or use observations from model outputs to inform analysis of functional groups in the FN. Third, adding just two terms to each target unit model rather than 73 (MG) or 175 (TY) terms reduces the likelihood of overfitting the model. Our final *kinematics + reachFN* model contained the trajectory features with 48 x-y-z velocity terms, three average position terms, and the coincident and leading network feature terms (Eq. 5). Note that we use the term *functional group* to denote subsets of the FN for which functional interactions were studied in the GLM (in the unmodified kinematics + reachFN model, the functional group is the entire set of source units to the target unit).

**Figure 3:**
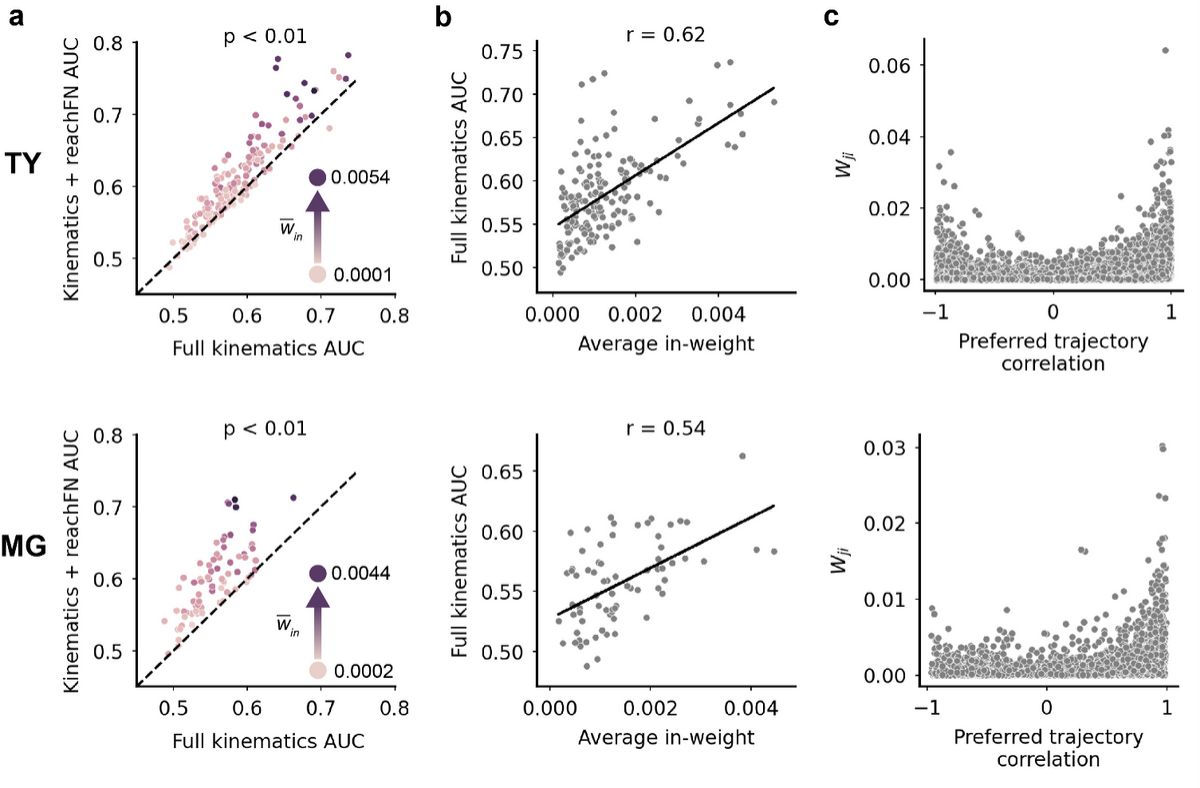
Network features improve the encoding model and first order FN structure is linked to kinematic tuning. (A-C) *Top*: monkey TY. *Bottom*: monkey MG. (A) Adding the reachFN network features to the full kinematics model improves prediction of single-unit activity (TY: p<0.01 by one-sided sign-test; MG: p<0.01). Each unit’s hue corresponds to average in-weight in the FN. (B) Performance of the full kinematics model (which contains no network feature terms) increases with average in-weight to the unit in the FN (TY: r = 0.62, Pearson correlation; MG: r = 0.54). (C) A scatterplot of all edge weights versus the pairwise preferred trajectory correlation.

The inclusion of network activity improves the model significantly across the population (TY: p < 0.01; MG: p < 0.01, one-sided sign test; Fig. 3a). Performance of the full kinematics and kinematics + reachFN models was similar across motor and somatosensory cortical areas (Supplementary Fig. 3a-b). We found that performance of the full kinematics model – which did not include network features – improved with increasing average in-weight in the functional group (TY: Pearson correlation r = 0.62; MG: r = 0.54; Fig. 3b). Additionally, model improvement from adding network features was positively correlated with average in-weight (TY: r = 0.60; MG: r = 0.62; Supplementary Fig. 3c). While we expected that a model incorporating functional group activity would exhibit larger performance gains with stronger inputs, it is striking that the units most strongly tuned to the full kinematics model (without network features) also received stronger functional group inputs. Unit pairs with strongly correlated preferred trajectories also tended to have strong functional connections (Fig. 3c). Importantly, the relationship between average full kinematics AUC in a pair of units and their preferred trajectory correlation was weak (Supplementary Fig. 3d), suggesting that even though model performance and trajectory correlation of units may not be strongly linked to each other, the strength of the connections between the units (weights) is independently associated with both better performance and more aligned preferred paths. None of these findings are the result of variations in unit firing rate or waveform signal-to-noise ratio (Supplementary Fig. 4). Taken together, these results demonstrate that functional interactions influence single-unit activity, that a unit’s tuning to kinematics increases with the degree to which it is interconnected with the surrounding network, and that strongly connected units exhibit similar (or to a lesser extent, opposite) tuning properties.

We note here that all GLMs were L2-regularized using the penalty weight (*α*) that maximized AUC on held-out test data, evaluated for models using lead and lags of [−100, + 300]ms and [−200, + 300]ms (Supplementary Fig. 5). Based on these results, we trained all kinematics models with *α* = 0.05 and all models incorporating the network with *α* = 1 × 10^−6^. We note that stronger penalty weights tended to reduce scaling coefficients for network terms in the kinematics + reachFN model and caused performance to drop to the level of the full kinematics model. This lends further evidence that functional interactions provide additional predictive power.

### The topology of strong functional interactions underlies accurate prediction of single-sample activity

**Figure 4:**
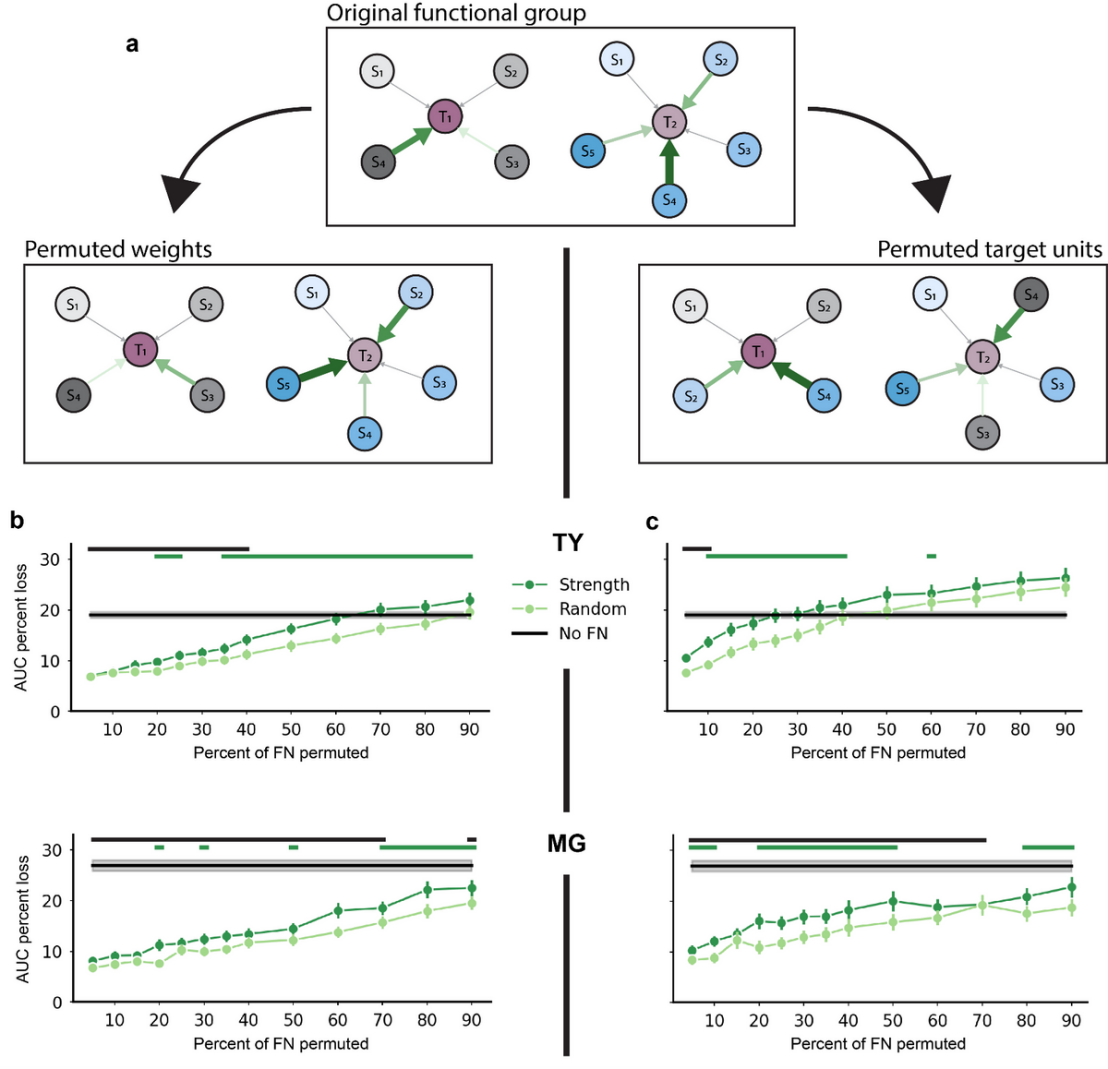
The topology of strong functional interactions underlies accurate prediction of single-sample activity. (A) Diagram of permutation methods. *Top*: two original functional groups of source units (S_n_) and their input edge weights to target units (T_n_). Green arrows indicate membership in the strongest N% of edge weights, with larger and darker arrows indicating a larger weight. Gray arrows constitute the remaining edges in the FN. *Lower-left*: for permuted weights, the strongest edges were permuted freely, even across functional groups, while the source-target unit pairs were unchanged. *Lower-right*: for permuted target units, the source-edge pair was held constant, but the target unit receiving input from the pair was permuted freely. This resulted in entirely new functional groups. Note that for simplicity this diagram does not depict the existence or permutation of reciprocal connections or the interconnectivity of functional groups. (B) *Top*: percent AUC loss on the training set (mean ± sem for 250 shuffles) resulting from permuting the strongest N% of weights (*dark green*) or a random N% (*light green*) versus the size of the permuted functional group for monkey TY. Green lines above the plot indicate functional group sizes for which permuting the strongest weights resulted in significantly greater loss than permuting random weights (p < 0.01, one-sided sign test). Black lines indicate group sizes for which the effect of removing the network feature terms entirely was significantly greater than permuting the strong weights (p<0.01). *Bottom*: same for monkey MG, with 500 shuffles. (C) Same as B for the effect of permuting target units in the original functional groups.

The finding that stronger average inputs correlated with stronger tuning to kinematics and to greater performance gains provided by network features (Fig. 3b and Supplementary Fig. 3c) could mean that single-unit prediction of activity relies simply on the total strength of functional inputs; alternatively, model performance may depend on the specific structure of the strong connections. Previous work in murine visual cortex has shown that the precise structure of strong connections in the FN are most informative of single-trial activity^8,34^. Following the methodology described in Kotekal and MacLean^8^, we investigated the importance of structured strong connections by selecting functional groups comprising N% of the strongest edges and manipulating these functional groups in one of two ways. Each edge in the network can be represented by its source unit (S_n_ in Fig. 4a), target unit (T_n_), and weight (denoted by arrow size and hue). Permuting edge weights amongst unchanged source-target pairs leaves the existing functional groups intact but alters the weight multiplier applied to source unit activity (Fig. 4a, bottom left). This manipulation tests the models’ reliance on the precise relationship between input activity and edge weight. Permuting the target unit, on the other hand, maintains the weight multiplier applied to source unit activity but changes the target unit receiving the input, thus changing the functional group of inputs to each single-unit model (Fig. 4a, bottom right). This manipulation tests the importance of target units receiving input from specific functional groups. After each manipulation, network features computed from the permuted FN were substituted into the kinematics + reachFN model. We did not re-train the GLM with manipulated network features, but rather computed the AUC loss on training data compared to the intact kinematics + reachFN model. Re-training the GLM would optimize model coefficients to minimize model error, which would obscure the effect of FN permutations. The AUC loss resulting from each manipulation was compared to the effect of an identical manipulation but of a randomly selected functional group of matched size and to the effect of completely removing network features from the model.

Across 250 train/test splits for monkey TY, permuting weights in the connection strength functional group resulted in significantly greater information loss than permuting weights randomly for functional group sizes of greater than or equal to 20% except for 30% (p < 0.01, one-sided sign test; Fig. 4b, top). The effect of removing FN terms entirely from the GLM was significantly greater than the effect of weight permutations for set sizes up to 40%, beyond which inclusion of weaker connections had a similar effect as removing the network features entirely. Across 500 permutations of weights for monkey MG, we found that the strongly connected functional group carried more information than the random functional for group sizes of 20%, 30%, 50% and 70–90% (p < 0.01; Fig. 4b, bottom left). The effect of removing the FN terms from the model significantly exceeded the effect of permuted weights for all but one functional group size – the 80% permuted FN (p < 0.01, one-sided sign test).

As with permuted weights, permuted target units produced higher AUC loss as group size increased. In contrast with permuted weights, strongly connected functional inputs to target units were particularly important for smaller group sizes. Disruption of strongly connected functional groups had a consistently larger effect than random permutation for group sizes of 10–40% and 60% in monkey TY and group sizes of 5–10%, 20–50%, and 80–90% in monkey MG (p < 0.01). The effect of removing the FN terms from the model significantly exceeded the effect of permuted target units for functional group sizes up to 10% in monkey TY and up to 70% in monkey MG.

These results demonstrate that prediction of single-unit activity depends on the precise topology of strongly connected functional groups rather than average in-weight alone. We also showed that disrupting the topology of strongly connected functional groups resulted in AUC loss comparable to complete removal of network features, suggesting that most available information was present in a subset of strong connections (this effect was most striking for permuted target units in monkey TY). Taken together, these results provide evidence that the specific structure of interactions captured by the FN are informative of single-unit activity in sensorimotor cortical populations, particularly for strongly connected functional groups.

### A context-specific functional group reorganizes during prey-capture

Although these results and prior work demonstrate the importance of strongly connected functional groups in the FN, previous work has also shown that the FN depended on the specific kinematics^7^. Consequently, we next evaluated how specific a FN was to prey capture, which we refer to as reach, as compared to a wide range of non-prey capture behaviors which we refer to as spontaneous. We trained a kinematics + spontaneousFN model by computing network feature terms from the dot product of the spontaneousFN with the spiking activity associated with reach trajectory samples. We then tested this model on network features computed with reachFNs to compare generalization of the kinematics + spontaneousFN model against the performance of the original kinematics + reachFN model. Essentially, we asked whether functional interactions computed during spontaneous behavior were informative during prey-capture. We observed that the kinematics + spontaneousFN model generalized well for most units – in fact, there was only a subset of the population for which it clearly could not generalize (TY = 40/175 units, MG = 9/73). We call this subset the *context-specific* functional group and the remaining units *context-invariant*. The classifier threshold separating the two groups was selected by identifying the kink in the plot of sorted AUC difference between the models (Supplementary Fig. 6) which corresponded to the point at which the median-filtered derivative of the sorted AUC difference fell below 7.5% of its maximum.

**Figure 5:**
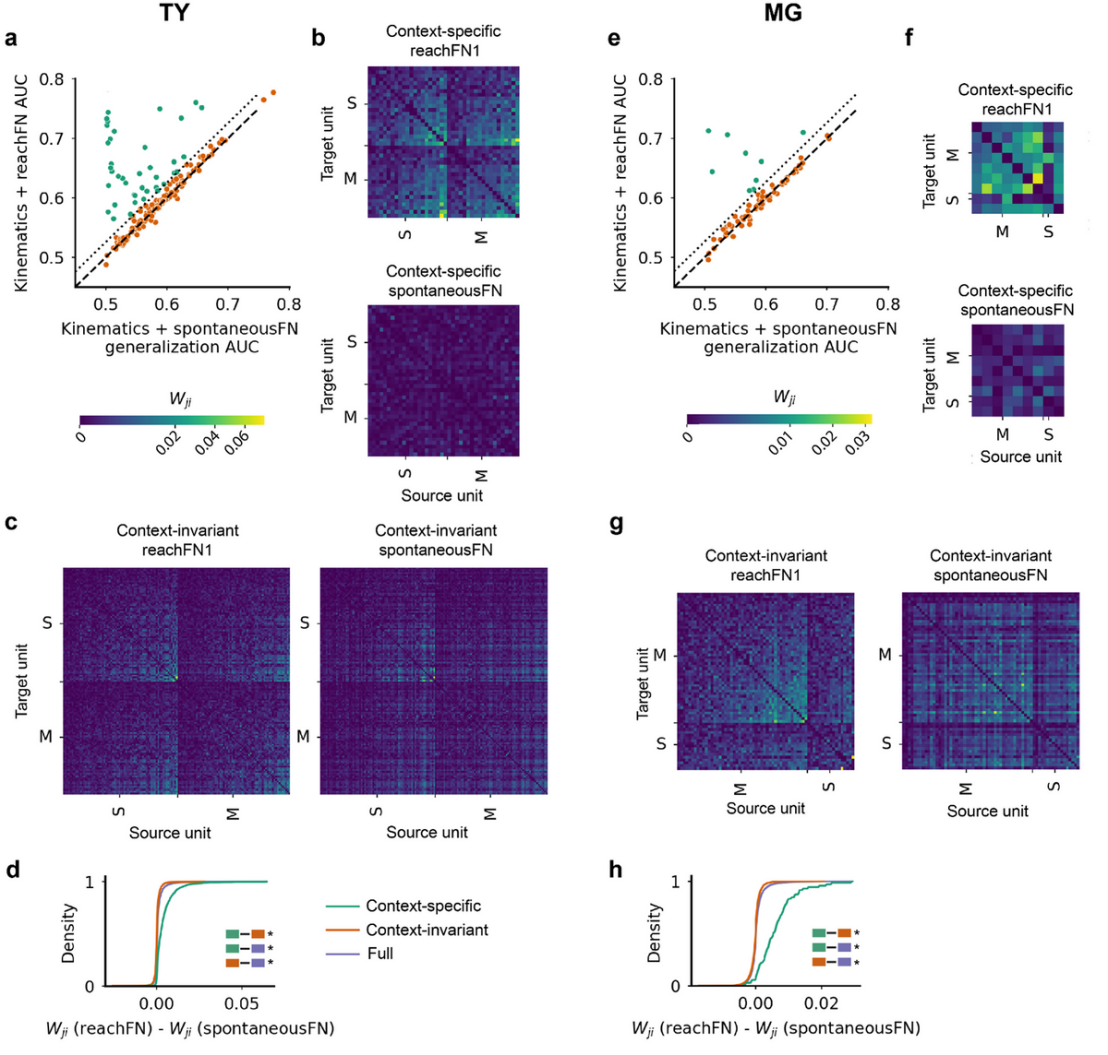
A context-specific functional group reorganizes during prey-capture. (A-D) Monkey TY. (A) The kinematics + reachFN model versus the network encoding model trained with spontaneousFN network features and tested on reachFN network features. Units above the dotted line are defined as the context-specific functional group (*green*, 40/175 units), and the remaining are context-invariant units (*orange*, 135/175 units). (B) The context-specific functional group, with reachFN1 on top and spontaneousFN on bottom. The color scale of edge weights is displayed under A. (C) The context-invariant reachFN1 and spontaneousFN. (D) The cumulative distribution of the difference in edge weights between reachFN1 and spontaneousFN for the context-specific, context-invariant, and full (*purple*) groups. Distribution comparisons are inset as colored pairs of boxes. The * indicates significantly different distributions with p<0.01 (two-sided median test). (E-H) Corresponding results for MG, with 9/73 units in the context-specific functional group.

We isolated the context-specific and context-invariant functional groups for reachFN1 and spontaneousFN (Fig. 5b–c,f–g). In monkey TY, we found that the edge-wise FN changes from spontaneousFN to reachFN1 were significantly different between the context-specific and context-invariant functional groups and between each functional group and the full FN (p < 0.01 for all comparisons, two-sided median test; Fig. 5d). The context-specific functional group skewed more toward an increase in weights, while the context-invariant group skewed toward decreasing weights. These effects were recapitulated in monkey MG (p < 0.01 for all comparisons; Fig. 5h) with the same directional effects. These and subsequent results are consistent for reachFN2 and are not reported for brevity.

**Figure 6:**
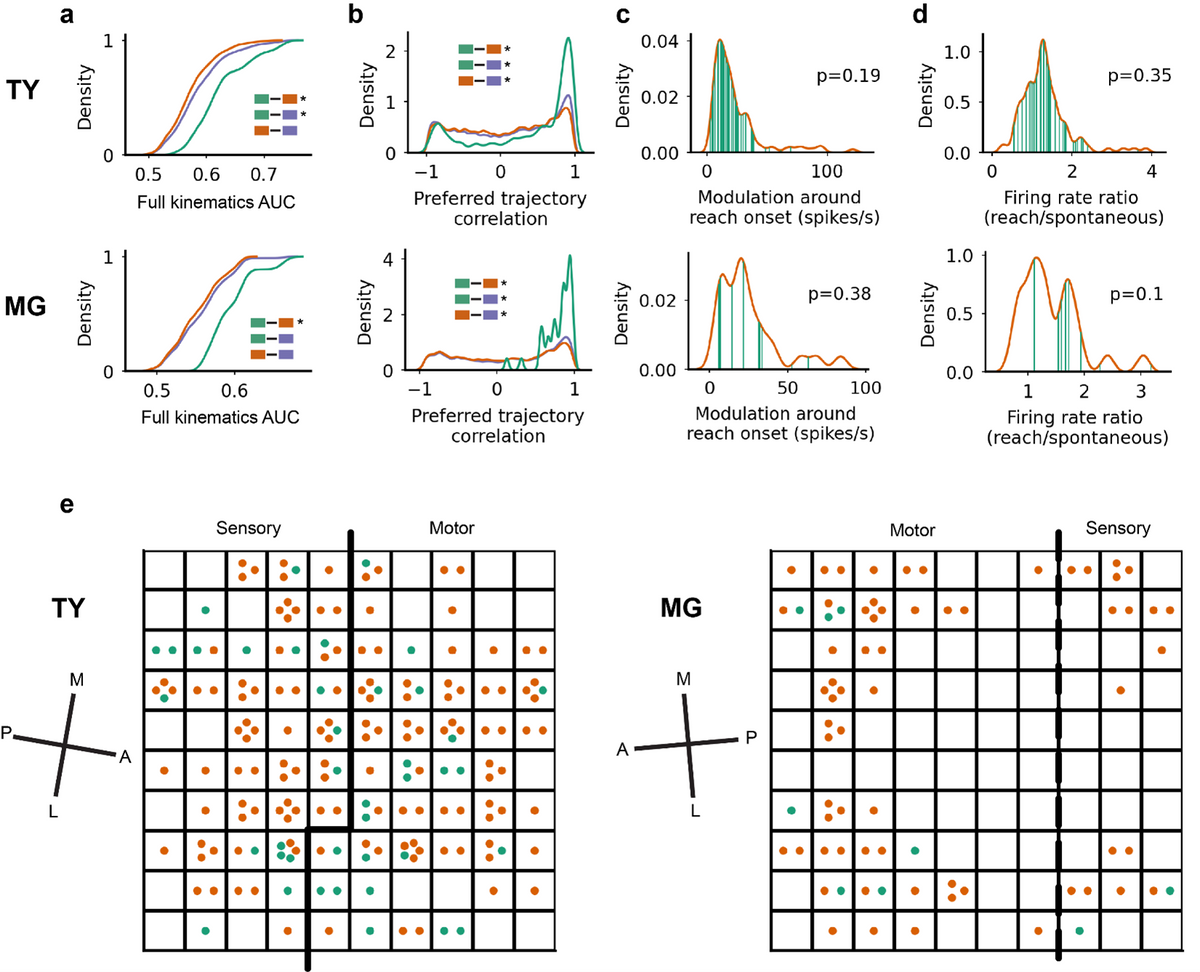
Tuning properties and cortical location of context-specific and context-invariant functional groups. (A) Cumulative distribution of AUC values in the full kinematics model for TY (*top)* and MG (*bottom*), separated by context-specific vs context-invariant/full groups. Legend and statistics are denoted as in Fig. 5, with the additional note that comparisons without * are not significant (p>0.05). (B) Distributions of preferred trajectory correlation. (C) Distribution of unit modulation at reach onset for the context-invariant functional group (*orange*) overlaid with the context-specific functional group (*green*), with p-values comparing these distributions. (D) Ratio of unit average firing rates during reaching over rates during spontaneous behavior. (E) Cortical location of context-specific and context-invariant units on the array. The dashed-line boundary between motor and sensory areas for monkey MG indicates less confidence in the precise location of the boundary compared to monkey TY (see Methods). The cardinal axes in cortex were estimated from surgical photos and are denoted medial (M), lateral (L), anterior (A) and posterior (P).

The context-specific functional group which reorganized during prey-capture was also more tightly linked to forelimb movement. We found that the full kinematics model more accurately predicted spiking activity for units in the context-specific functional group than for context-invariant units (p < 0.01, TY and MG, two-sided median test; Fig. 6a), as well as the full set of units in monkey TY (p < 0.01) but not MG (p > 0.05). Preferred trajectories were more strongly and positively correlated within the context-specific functional group than within the context-invariant or full FNs (p < 0.01; Fig. 6b), while the context-invariant functional group skewed toward weaker and negative correlations (p < 0.01 compared to the full FN). The shift toward strong positive trajectory correlations in the context-specific group appears to be caused by preferential inclusion of units tuned to extension movements (Supplementary Fig. 2b-c). The results shown in Figs. 5 and 6 were not a result of either increased modulation around reach onset (Fig. 6c) or different ratios of average firing rates during reaching versus spontaneous behavior (Fig. 6d) in the context-specific functional group. We note that for Fig. 6c–d we compared *AUC-matched* functional groups, meaning that we used only the units in the context-invariant group with full kinematics AUC exceeding the lowest AUC in the context-specific group. We did this to ensure a fair comparison, given that the modulation and firing rate ratio both correlated weakly with full kinematics AUC (r = 0.13 and r = 0.20, respectively, not shown). Finally, we examined the location of context-specific and context-invariant units on the cortical map and found that members of each functional group were spread across estimated motor and somatosensory areas (Fig. 6e). For both monkeys, the majority of units in the context-specific group were recorded from channels also recording context-invariant units, confirming that functional group membership was not the consequence of channel differences.

To rule out the possibility that results from Figs. 5 and 6 might be explained simply by the higher distribution of AUC values in the context-specific functional group, we repeated some analyses using AUC-matched sets. To do so, we included all pairs of units within the context-invariant and full FNs for which both units had full kinematics AUCs exceeding the lowest full kinematics AUC in the context-specific functional group. AUC-matched comparisons of preferred trajectory correlations (Supplementary Fig. 7a) and edge-wise weight differences (Supplementary Fig. 7b) recapitulate the results shown in Fig. 6a and Fig. 5d,h, respectively, that compared unmatched distributions. Finally, the context-specific functional group did not comprise all the units performing well in the full kinematics or kinematics + reachFN model but were intermixed with well-explained context-invariant units (Supplementary Fig. 7d).

## Discussion

We have shown that precisely timed single-unit activity during forelimb movements can be predicted accurately by an encoding model incorporating functional interactions, a temporally-extended hand velocity trajectory, and the average hand position taken over the trajectory. We also demonstrated that tuning to kinematics depends fundamentally on functional interactions between units – particularly on structured strong connections. This builds on past work in macaque motor cortex demonstrating that movement-related information is present in pairwise spike count correlations^5,6^, and provides complementary insights to recent work showing that the structure of fine-timing spike correlations in a FN contains movement-related information and evolves systematically over the course of behavior^7^. Finally, we identified a context-specific functional group within which functional interactions reorganize to produce the natural forelimb movements during prey capture.

Given evidence that neural activity recorded in association with highly constrained and over-trained tasks may not generalize completely to naturalistic, unrestrained behavior^13,17,18,28^, it was not guaranteed that the results of our full kinematics model would match those from a planar reaching task^14^. However, as in that study, we found that the trajectory model predicted single-unit activity more accurately than a velocity model and that the full kinematics model was most accurate for trajectories including a range of lead and lag kinematics. In fact, the same [−100, + 300]ms model that performed best for M1 units in macaques executing a random-target pursuit task was also amongst the best-performing models here. We show that the model’s accuracy extends beyond M1 to predict units across sensorimotor cortex; this aligns with studies demonstrating similar encoding^35^ and decoding^36^ of distal limb movements of the wrist and digits for units in M1 and area 3a. We also found that significantly tuned units exhibited distinct preferred trajectories in addition to average position tuning. Since the trajectory tuning model extended to naturalistic behavior, it served as a useful foundation for investigating the additional information provided by the functional network.

Inclusion of network features in the kinematics + reachFN model significantly increased predictive power over the full kinematics model, and performance of the full kinematics model increased with stronger average functional inputs from other units – despite no direct link built into the full kinematics model. Furthermore, we demonstrated that prediction of single-unit activity depends on the precise topology of strongly connected functional groups rather than average in-weight alone. This agrees with a similar study in murine visual cortex which demonstrated that the topology of the functional group containing the largest 25% of edge weights was critical to the performance gained by incorporating network features^8^. We also showed that all the information provided by network features could be eliminated by disruptions to the topology of strongly connected functional groups. For monkey TY, information was concentrated in the strongest 10% of functional inputs and the strongest 40% of strong weights. For monkey MG, information was concentrated in the strongest 70% of both weights and functional inputs.

Work by Levy et al.^9^ showed that both tuned and untuned units in visual cortex play essential roles in the FN, and that untuned units were central to the structure of the network. This is in contrast with two results presented here: that strongly interconnected units tended to be more tuned to kinematics, and that members of the context-specific functional group were both more strongly connected to each other and more tightly linked to kinematics. This suggests that untuned units may play a different role in sensorimotor cortex than in visual cortex, which is consistent with the finding that areas and behaviors with different computational constraints exhibit distinct population dynamics^37,38^. We posit that this difference is related to the generation of temporally smooth population dynamics that are necessary for production of motor behavior^3,4,12^.

We identified a subset of the population, the context-specific functional group, for which the kinematics + spontaneousFN model could not generalize to match the kinematics + reachFN model when the animal engaged in prey-capture reaching. Surprisingly, the context-specific group comprised less than 25% of the population, while interactions measured during spontaneous behavior generalized well to explain interactions during prey-capture for the remaining units. When we compared the context-specific group to the context-invariant and full groups, we discovered that the context-specific functional group was more strongly interconnected in the reachFN, contained pairs of units with more positively correlated preferred trajectories, and reorganized its connectivity patterns significantly between the spontaneousFN and reachFN. The structure of interactions between context-invariant units was comparatively consistent across spontaneous and reaching behavior. Additionally, the context-specific functional group was more strongly tuned to forelimb kinematics than the context-invariant group.

The simplest explanation for the context-specific functional group is that it plays a differential role in extension movements. We showed in Fig. 1b that much of the population exhibited higher firing rates during extension of the hand into the prey-capture workspace and showed in Supplementary Fig. 2b that the context-specific functional group was dominated by units tuned to extension. It is likely that dynamic extensions of the hand – and the muscle activations involved in such movements – are overrepresented during prey-capture but comprise a smaller proportion of spontaneous behavior. Grasping often occurred coincidently with hand extension, raising the possibility that many units including those in the context-specific functional group were tuned to grasp as well as hand extension (although grasp was not quantified by DLC pose estimation in this study). Future work might move beyond a single broad class of spontaneous behavior, which spans the marmoset’s natural behavioral repertoire. Instead, we could apply pose estimation to spontaneous behavior, identify distinct behavioral classes, and investigate structured interactions and functional groups underlying each class. If the context-specific functional group is, in fact, part of a neuronal module to produce reach-to-grasp movements, we would expect to find that FNs computed during behaviors involving such movements would match the reachFN closely – and vice versa for other behaviors.

Work by Dann et al.^39^ showed that modularity in the functional network links large groups of interconnected units in a single cortical area to smaller groups in other areas, suggesting a mechanism for information flow between areas. It could be that members of the context-specific functional group described here, which span motor and sensory regions and are tightly coupled by preferred trajectory correlations and strong edge weights, participate in flexible modules to facilitate inter-area communication.

The differences between the context-specific and context-invariant groups aligns with recent work demonstrating that reliable pairwise correlations, rather than first-order statistical features of spike trains, are the building blocks of coding in visual cortex^10^. The reorganization of the context-specific functional group, which was strongly tuned to kinematics, demonstrates a link between precisely structured interactions and kinematic encoding in sensorimotor cortex.

An alternative (and more speculative) interpretation of the context-specific and context-invariant functional groups is that they might be differentially involved in processes identified by the population dynamics framework. The functional interactions making up the context-invariant group were relatively consistent across reaching and spontaneous motor behaviors in the home enclosure. It is possible that these stable pairwise interaction patterns could preferentially contribute to the generation of low-dimensional and rotational dynamics that evolve in a predictable fashion with low-tangling^12^. The context-specific group, on the other hand, may contribute to deflections in the neural trajectory correlated with muscle activity^12^ or to moving the fixed point about which rotational dynamics unfold in neural space^40^. In that framework, the position of the fixed point determines the angle of rotations – which unfold at a conserved frequency – and varies systematically with direction of movement, suggesting a link between classical tuning and population dynamics. Similarly, the context-specific functional group presented here was strongly tuned to kinematics. It is important to note that no studies in the dynamical systems framework have identified distinct subsets of the population that contribute differentially to separate dimensions or features. This may mean the context-specific and context-invariant functional groups do not, in fact, map directly onto features identified by this approach. On the other hand, previous dynamical systems work studied constrained and over-trained motor behaviors that may exhibit different neural activity patterns than those during naturalistic movements used in the current work^18,41^. Behaviors in those studies span a smaller and repeated range of speeds, postures, and amplitudes; do not rely on continuous online adjustments to track evasive targets; and are externally cued with instructed delay periods rather than internally cued by ongoing motivation for capture of live prey. Furthermore, prior work did not use neural recordings from rich spontaneous behavior as a comparison with goal-directed behavior. Thus, future work to investigate the impact of the context-specific and context-invariant functional groups on population dynamics requires data suitable for all relevant contexts. This work should span spontaneous behavior, naturalistic forelimb movements, and trial-based reaches, and include simultaneous recordings of muscle activity. It may be that a different functional group is engaged preferentially for each context as well as class of spontaneous behavior; alternatively, structured interactions within the context-invariant group might be conserved across behaviors while the context-specific group reorganizes based on the demands of each behavior. The latter finding might illuminate possible links between the distinct functional groups and dynamical systems features.

## Methods

### Subjects

These experiments were conducted with two common marmosets, *Callithrix jacchus* (a 10 year old, 370g male designated TY and an 8 year old, 350g female designated MG). All methods were approved by the Institutional Animal Care and Use Committee of the University of Chicago.

### Data Collection - Behavior and Cameras

A custom-built, modular apparatus designed for prey-capture and other goal-directed tasks^42^ was attached at the top of the home enclosure. Subjects were allowed to enter the apparatus and engage in prey-capture voluntarily. When the subject was prepared and alert, the experimenter dispensed a single moth into the apparatus to initiate a prey-capture episode. The next prey was dispensed when the previous prey was either captured and eaten or had escaped the apparatus. The subject was engaged in spontaneous behavior in the home enclosure or in the apparatus when not directly alert and responsive to the prey-capture task. Spontaneous behavior included but was not limited to: leaping, hanging, grooming of self or partner, ambulating or resting in the home enclosure and visual exploration, non-reaching movements, or fine manipulation of prey in between capture episodes in the apparatus. A recording session lasted approximately ~ 1.5 hours for TY and ~ 2.5 hours for MG.

High-speed cameras (Blackfly S, 200 frames s − 1, 1440×1080 resolution; Teledyne FLIR) were used to record video for pose estimation by DLC. For marmoset TY, two cameras were positioned to optimize visibility of the left upper limb and recorded at 150 fps. For marmoset MG, five cameras recorded at 200 fps – two cameras for each side view and one front-facing camera to improve coverage for reaches occluded in the side views. Image acquisition was triggered by the marmoset activating an infrared beam-break sensor when approaching the partition within the apparatus. Additional cameras were used to record behavior in the home enclosure from a wider angle, but we do not attempt to classify or quantify behavior in the home enclosure in this work.

### Data Collection - Neural Recording

Each subject was implanted with a 96-channel Utah Array (Blackrock Microsystems, Salt Lake City, UT) using stereotaxic coordinates^43^ to target the forelimb area of the primary motor cortex in the right (TY) or left (MG) hemisphere. The surgical procedure is described in detail by Walker et al.^21^ Neural data was collected using a Blackrock Cereplex Exilis, which houses a digital amplifier, wireless transmitter and Li-ion rechargeable battery capable of powering ~ 90 minutes of continuous recording in a compact headstage. A quick-connect solution designed in-house facilitated the removal/attachment and charging cycle with minimal experimenter intervention and disruption to the marmosets’ natural behaviors (see Walker et al.^21^, which describes many of the design concepts which were adapted for the Exilis headstage). Additional battery life was provided in the MG recordings by a detachable external battery circuit designed in-house. Data was transmitted to 8 receiving antennas, then processed by additional products from Blackrock Microsystems. All data was recorded as raw 30 kHz continuous signals.

### Data Collection - ICMS and receptive field mapping

The extent of motor and sensory areas on the TY array were estimated from results of intra-cortical microstimulation (ICMS) and receptive field mapping of tactile and proprioceptive feedback, which were conducted at night during quiet restfulness. For ICMS, a low current was used to identify muscle groups for which stimulation evoked movement. Then the current was reduced to identify the specific muscle target of the channel and the lowest current that evoked a response. For receptive field mapping, the skin was lightly brushed with a cotton-tipped applicator (tactile) or the muscle body was palpated (proprioceptive). This was repeated 20 or more times for each muscle/body region using a 5sec on/5 sec off protocol. Peri-stimulus time histograms were computed from the repetitions and mapped onto the array for manual inspection. Cortical area boundaries were estimated by comparing the composite maps of ICMS and receptive field mapping to prior cortical mapping results in marmosets^43–46^.

Only receptive field mapping was completed for the MG array. The boundary between motor and sensory areas was estimated based on these results and by comparison to the combined mapping results of the TY array. We have noted the lower degree of confidence in the exact boundary with a dotted line in Supplementary Fig. 3 and Fig. 6. We note that the precise location of the boundary does not affect any results and is only used for display purposes on array maps, raster plots, and functional network connection matrices.

### Data Processing - Spike Sorting

Spike sorting was performed on raw neural data at 30 kHz using Spike Interface^47^. Ironclust (https://github.com/flatironinstitute/ironclust) was the primary sorter, with SpykingCircus^48^ and waveclus^49^ used to cross-reference for consistent units that were identified across all sorters. Inter-spike interval and signal-to-noise ratio thresholds (ISI violation rate < 0.5, SNR > 5) were applied to automatically pre-classify units as multi-unit activity, then units identified by all three sorters were automatically classified as well-isolated single units. All automatically sorted spikes were manually curated using phy (https://github.com/cortex-lab/phy).

### Data Processing - Kinematics

A DeepLabCut network (DLC)^19^ with Resnet-50 base architecture was trained on 2,343 labeled images from TY and MG recording sessions. Three labels were applied to each side of the wireless headstage housing (for head-tracking in future work), three to the upper limb on each side (shoulder, elbow and wrist), and three to corners of the apparatus to establish a coordinate system - a total of 15 labeled points. The model was iteratively trained and refined until pose estimation was consistent throughout all significant upper-limb movements in the prey capture workspace. Anipose was used to apply the DLC network to videos and to perform subsequent 2D filtering and 3D calibration and triangulation, as described in Moore et al.^22^ and Karashchuk et al.^20^

We added multiple steps of post-processing to reduce the effect of brief tracking lapses in outputs from the well-trained DLC network (such as spurious jumps and brief occlusions). Each step was applied independently to all markers and video events. We first filtered out timepoints with reprojection error greater than 20 pixels (35 for MG) and fewer than two cameras tracking the label, leaving only well-tracked segments of kinematic data. We removed very brief segments shorter than 50ms, then filled tracking data back in for inter-segment gaps shorter than 200ms (these steps in conjunction eliminated epochs dominated by poor tracking but containing intermittent, brief high-likelihood tracking which often caused problems during interpolation). Most lapses were fixed or removed correctly after these simple steps. Next, we removed any remaining marker jumps using the reprojection error and position data together. Then we replaced any brief inter-segment gaps that remained with either original tracking data – if that data matched a linear interpolation closely – or with the interpolation. Finally, we trimmed long segments with a high percentage of interpolated data at the beginning and end of video events - these segments corresponded to the marmoset maintaining an occluded posture in the back of the apparatus before or after prey-capture. The processed data was smoothed with a 3rd order, 70ms Savitsky-Golay filter. We identified reaching segments by finding y-position peaks that indicated extension of the hand into the prey-capture space and assessing when hand speed crossed a threshold before and after each peak.

The continuous position of the shoulder marker was subtracted from hand marker position to isolate hand movements from postural changes, then differentiated to obtain the isolated hand velocity.

### Trajectory encoding model

To build the encoding model for each neuron, we used a generalized linear model that estimated the set of coefficients to maximize the likelihood of correctly predicting the spike count within a 10ms spike sampling window given the corresponding sample of kinematics. Each kinematic sample was the monkey’s hand velocity trajectory and average position over a kinematic sampling window of length $\tau_{lead}+\tau_{lag}$, with the kinematic and paired spike samples centered at $t_{0}$ . Samples of spikes and kinematics were extracted every 30ms throughout reaching segments, resulting in around 8,000 samples for monkey TY (8,149 for the [−100, + 300]ms model) and 4,000 samples for monkey MG (4,250 for the [−100, + 300]ms model). Instantaneous velocity trajectories were down-sampled to 40 Hz based on the observation that the power spectrum of velocities mostly fell below 25 Hz. Each velocity sample can be formalized as:

(Eq. 1) $${\hat{v}}_{t_{0}}=\left(\vec{v}\left(t_{0}-\tau_{lead}\right),\ldots ,\vec{v}\left(t_{0}+n\Delta t\right),\ldots ,\vec{v}\left(t_{0}+\tau_{lag}\right)\right)$$

where $\vec{v}\left(t_{0}+n\Delta t\right)$ is the instantaneous 3-dimensional velocity vector at time $t_{0}+n\Delta t$ and Δt=25ms. The full kinematics model relates these terms and the average position vector $\vec{p}$ to the conditional spike intensity of target unit i:

(Eq. 2) $$P\left(spike_{i}\left(t_{0}\right)|{\hat{v}}_{t_{0}},\vec{p}\right)=exp\left(\gamma +\vec{k}•{\hat{v}}_{t_{0}}+\vec{c}•\vec{p}\right)$$

where $\vec{k}$ is referred to as the preferred velocity trajectory of the neuron and $\vec{c}$ is the vector of coefficients for the average position terms. The preferred path, or *pathlet*, of the neuron is obtained by integrating $\vec{k}$ in time as if it were a vector of 3-dimensional velocities.

GLMs were implemented using the Python statsmodels package and fit with L2-regularization. We trained all kinematics models with penalty weight α=0.05 and all models incorporating the network with $\alpha =1\times 10^{-6}$.

### Area under the receiver operating characteristic curve

To assess the predictive power of each encoding model, we computed receiver operating characteristic (ROC) curves that quantified the relationship between the probability of correctly predicting a spike (hit probability) versus the probability of incorrectly predicting a spike when it was absent (false positive probability). We used 80% of spike-kinematics sample pairs to train the model for each unit and used 20% as held-out test data. We predicted the conditional spike intensity from the encoding model given the 20% of held-out kinematics samples and compared predictions to the held-out spike samples using a set of thresholds to predict spikes when the conditional spike intensity crossed the thresholds. We then computed the area under the ROC curve (AUC), which measures the predictive value of the model and can vary from 0.5 (no predictive power) to 1.0 (perfect prediction). The train/test split was sampled randomly 500 times with replacement, resulting in 500 encoding models for each unit.

### Functional Network computation

We created weighted, directed FNs by computing pairwise spike time statistics between recorded units. We binned the recorded spike trains into 10ms bins, assigning a value of 1 if at least one spike occurred in that bin, and 0 otherwise. We then computed the confluent mutual information (conMI) between the binned spike trains^30^. ConMI quantifies the information about the firing state of target unit i at time t or t+1 that is gained from knowledge of the firing state of a source unit j at time t:

(Eq. 3) $$w_{ji}=conMI_{ji}=\sum_{j(t)\in \{0,1\}}\;\sum_{i(\overset{ˆ}{t})\in \{0,1\}}\;p(j(t),i(\overset{ˆ}{t}))•{log}_{2}\left[\frac{p(j(t),i(\overset{ˆ}{t}))}{p(j(t))∙p(i(\overset{ˆ}{t}))}\right]$$

(Eq. 4) $$wherei(\overset{ˆ}{t})=\left\{\begin{array}{c} 1,ifi\;(t)=1ORi\;(t+1)=1\\ 0,\;otherwise \end{array}\right.$$

We computed reachFN1 from half of the reaching segments chosen at random and reachFN2 from the other half. We paired the kinematics from reachSet1 with the FN computed during reachSet2, and vice versa, to eliminate the concern that kinematics and the FN features might co-vary within a single sample. We also computed the FN during the remainder of the session in which the marmoset was behaving in an undirected, spontaneous manner either in the apparatus or the home enclosure (spontaneousFN). Each FN was represented as a square matrix of directed edge weights (the conMI) between nodes (units), with target units represented along the rows and source units along the columns.

### Incorporating the functional network into the encoding model

Activity in the functional group was incorporated in the encoding model by computing the sum of input weights times spiking activity:

(Eq. 5) $$P\left({spike}_{i}\left(t_{0}\right)|{\overset{ˆ}{v}}_{t_{0}},\overset{⇀}{p},F^{0},F^{1}\right)=exp\left(\gamma +\overset{⇀}{k}•{\overset{ˆ}{v}}_{t_{0}}+\overset{⇀}{c}•\overset{⇀}{p}+\beta_{0}F^{0}+\beta_{1}F^{1}\right)$$

(Eq. 6) $$F^{0}=\sum_{j}\;w_{ji}s_{j}^{0}andF^{1}=\sum_{j}\;w_{ji}s_{j}^{1}$$

where $w_{ji}$ is the edge weight (the conMI value) from source unit j to target unit i, and $s_{j}^{0}$ and $s_{j}^{1}$ are spike activity in the source unit at times t and t-1, respectively. Thus, $F^{0}$ and $F^{1}$ are the coincident and leading network features and $\beta_{0}$, $\beta_{1}$ are the corresponding scaling terms fit in the GLM.

### Statistical Tests

We used three statistical tests to evaluate significance of results. We used the sign test for paired tests, including comparisons of unit performances in two encoding models and comparing FN permutations. We used the median test to compare the medians across distributions for context-specific, context-invariant and full FNs. We chose the median test because it is conservative and valid for distributions with different sample sizes and variance. We used the Pearson correlation to quantify preferred trajectory correlations and to evaluate the correlation between pairs of features (for example, full kinematics AUC vs average in-weight). For correlations, we considered |r|<0.2 to be uncorrelated, |r| in [0.2, 0.5] to be weakly or moderately correlated, and |r|>0.5 to be strongly correlated.

## Data Availability

Data from marmoset MG is available at INSERT_LINK**. Data availability for marmoset TY is delayed due to the dataset’s use in manuscripts currently in preparation. ** This dataset will be deposited on DANDI and the link inserted here prior to final acceptance.
